# From Concept to Completion: Strategies for Engaging Older Adults and Communities in Behavioral Clinical Trials

**DOI:** 10.1093/geroni/igaf122.855

**Published:** 2025-12-31

**Authors:** Ryan Mace, Kathi Heffner

## Abstract

Behavioral clinical trials increasingly emphasize community engagement to enhance recruitment, retention, and intervention effectiveness, particularly for underserved populations. However, translating community partnerships into successful trials presents challenges requiring innovative approaches. This symposium aligns with the GSA 2025 theme, “Innovative Horizons in Gerontology,” by showcasing strategies for integrating older adult communities into behavioral clinical trials, from initial planning to large-scale implementation. Presenter 1 will discuss the process of engaging the Massachusetts Councils on Aging (MCOA; 350+ senior centers) in the development of a comparative effectiveness trial of Cognitive Behavioral Therapy with Social Enhancement (CBT-SOUL) versus social prescribing to reduce loneliness and social isolation in low-income and minoritized older adults. Presenter 2 will examine strategies for community partnership with MCOA from planning through execution of a feasibility RCT testing My Healthy Brain, a mindfulness-based dementia prevention intervention for older adults with subjective cognitive decline, supported by an NIA K23. Presenter 3 will present community engagement strategies for the Hoping & Coping project, an NCCIH K23 and RCMAR study culturally tailoring and testing the feasibility of mindfulness-based cognitive therapy (MBCT) for Black older adults with chronic pain, depression, and early cognitive decline. Presenter 4 will discuss the transition from an NCCIH R61 feasibility trial to an R33 effectiveness trial of a mind-body walking group intervention delivered in community clinics for English- and Spanish-speaking adults with chronic pain. The Discussant will synthesize these presentations, highlighting methodological innovations in community engagement and the role of behavioral trials in advancing gerontology research. Behavioral Interventions for Older Adults Interest Group Sponsored Symposium

